# A longitudinal multimodal big data infrastructure for precision poultry monitoring

**DOI:** 10.3389/fdata.2026.1821612

**Published:** 2026-05-11

**Authors:** Daniel Essien, Yashan Dhaliwal, Suresh Neethirajan

**Affiliations:** 1 Faculty of Computer Science, Dalhousie University, Halifax, NS, Canada; 2 Faculty of Agriculture, Dalhousie University, Truro, NS, Canada

**Keywords:** agricultural data systems, big data infrastructure, data synchronization, longitudinal dataset, multimodal data integration, precision poultry farming, sensor fusion

## Abstract

Livestock systems are increasingly instrumented with heterogeneous sensors, yet the resulting data remain fragmented, short-lived, and rarely documented as integrated infrastructures. This gap limits the development of robust multimodal artificial intelligence under real production conditions. Here we present a longitudinal multimodal data infrastructure for poultry monitoring, spanning 22 consecutive weeks across five commercial-style barns. The dataset combines continuous RGB video (1080 p, 30 fps), continuous audio (48 kHz), periodic radiometric thermal imaging, and twice-daily environmental measurements, yielding 10.2 terabytes of temporally heterogeneous data. Rather than focusing on a specific predictive task, the study addresses the underlying data-engineering challenge: how to acquire, synchronize, store, and preprocess multimodal streams at production scale. We detail a reproducible system architecture for distributed sensing, local buffering, secure transfer, and cloud-based organization, together with standardized preprocessing pipelines for illumination correction, acoustic denoising, and radiometric temperature extraction. Temporal alignment is achieved through timestamp-based normalization across asynchronous modalities, with explicit characterization of alignment granularity and missing data under real-world constraints. This work positions multimodal livestock sensing as a data-systems problem. The resulting dataset supports longitudinal analysis, cross-modal querying, and the development and evaluation of machine learning and multimodal fusion approaches at appropriate temporal scales. By releasing both data and workflows, we provide a transparent and extensible foundation for building and evaluating AI systems in precision agriculture.

## 1 Introduction

The increasing digitization of livestock systems has accelerated the adoption of machine learning for monitoring animal health, welfare, and productivity. In poultry systems, sensing approaches have been applied to detect welfare states and physiological anomalies using individual modalities such as acoustic analysis (Manikandan and Neethirajan, 2025; Qiao et al., 2026), computer vision (Nasiri et al., 2024), infrared thermography (Solis et al., 2024), and environmental sensing. While these unimodal systems can achieve strong predictive performance within constrained settings, they capture only partial representations of complex biological and environmental processes.

Multimodal integration offers a path toward more comprehensive monitoring by combining complementary sensor streams through data fusion architectures. However, despite growing interest in multimodal learning for animal systems, most published studies integrate a limited subset of modalities and operate under short-duration or tightly controlled experimental conditions (Sun et al., 2025). As a consequence, reproducibility, cross-study comparability, and real-world deployment remain limited. Beyond algorithmic development, a more fundamental bottleneck persists at the level of data infrastructure.

Large-scale multimodal datasets suitable for longitudinal analysis in commercial-style agricultural environments remain scarce. Existing animal-related datasets such as COCO Animal (Lin et al., 2015), Animal-POSE (Cao et al., 2019), and AP-10K have advanced computer vision research, yet focus primarily on static RGB imagery collected in uncontrolled outdoor contexts. Recent efforts have introduced multimodal datasets such as MMCows (Choi et al., 2024) which synchronizes wearable sensors with Multiview video and MetaWild (Li et al., 2025) which pairs visual data with environmental metadata for re-identification. However, our dataset is distinguished by its longitudinal 22 week scope and a broader array of modalities including continuous high-resolution audio and radiometric thermal images. Domain-specific datasets in cattle (Choi et al., 2024; Zia et al., 2023), swine (Chae et al., 2025), and broilers are typically restricted to single modalities or short-term recordings. Few datasets simultaneously address temporal continuity, heterogeneous sensor integration, and reproducible documentation across extended production cycles.

From a Big Data systems perspective, multimodal livestock monitoring introduces challenges that extend beyond model performance. Continuous sensing across video, audio, thermal, and environmental channels generates heterogeneous streams with divergent sampling rates, file formats, and storage demands. These streams must be temporally synchronized, securely transferred, archived at scale, and prepared for downstream analysis while preserving data fidelity. Real-world agricultural environments further introduce veracity constraints including noise, occlusion, illumination variability, and manual logging artifacts (Essien and Neethirajan, 2025; Panagi et al., 2025). The absence of openly documented acquisition frameworks and longitudinal datasets limits progress in scalable, deployment-ready AI systems for agriculture.

To address these infrastructural gaps, we present a longitudinal multimodal data acquisition framework and publicly release a high-volume dataset collected over 22 consecutive weeks across five commercial-style poultry barns. The dataset integrates four complementary modalities: continuous RGB video at 1080 p resolution, continuous 48 kHz audio recordings, periodic radiometric thermal imaging, and twice-daily environmental measurements of temperature and relative humidity. In total, the raw dataset comprises 10.2 terabytes of synchronized multimodal data spanning early to mature production phases.

This paper makes four primary contributions. First, we design and implement a scalable multimodal acquisition architecture capable of managing continuous heterogeneous sensor streams under commercial barn conditions. Second, we release the complete dataset and associated preprocessing pipelines through open repositories to support reproducibility and reuse. Third, we document modality-specific preprocessing workflows addressing illumination correction, acoustic denoising, and radiometric temperature extraction, enabling standardized downstream analysis. Fourth, we provide a systems-level characterization of the dataset through the lens of Big Data properties including volume, variety, velocity, veracity, and value, with explicit consideration of scalability and deployment constraints aligned with SDG 9.

Collectively, this work establishes a reusable data infrastructure for multimodal machine learning in precision livestock systems. Rather than advancing a specific predictive model, the focus is on enabling robust integration, temporal alignment, and reproducible dataset management at production scale. By documenting acquisition, synchronization, and storage strategies alongside open data release, this study provides foundational infrastructure for future research in sensor fusion, longitudinal modeling, and AI-driven agriculture.

The remainder of this paper is structured as follows. Section 2 describes the experimental environment and data collection protocol. Section 3 details the multimodal acquisition architecture and deployment configuration. Section 4 outlines preprocessing and data management pipelines. Section 5 characterizes the dataset through Big Data properties and descriptive statistics. Section 6 discusses infrastructural challenges, scalability considerations, and limitations. Section 7 concludes with implications for multimodal data systems in precision agriculture.

## 2 Experimental design and data collection protocol

### 2.1 Study site and barn configuration

Data collection was conducted at the Atlantic Poultry Research Centre, Dalhousie University Agricultural Campus, Truro, Nova Scotia, Canada. The facility comprised five enclosed research barns designed to reflect commercial-style poultry housing conditions. Each barn consisted of a single-room layout with defined functional zones for feeding, drinking, resting, and general movement.

**Figure 1:**
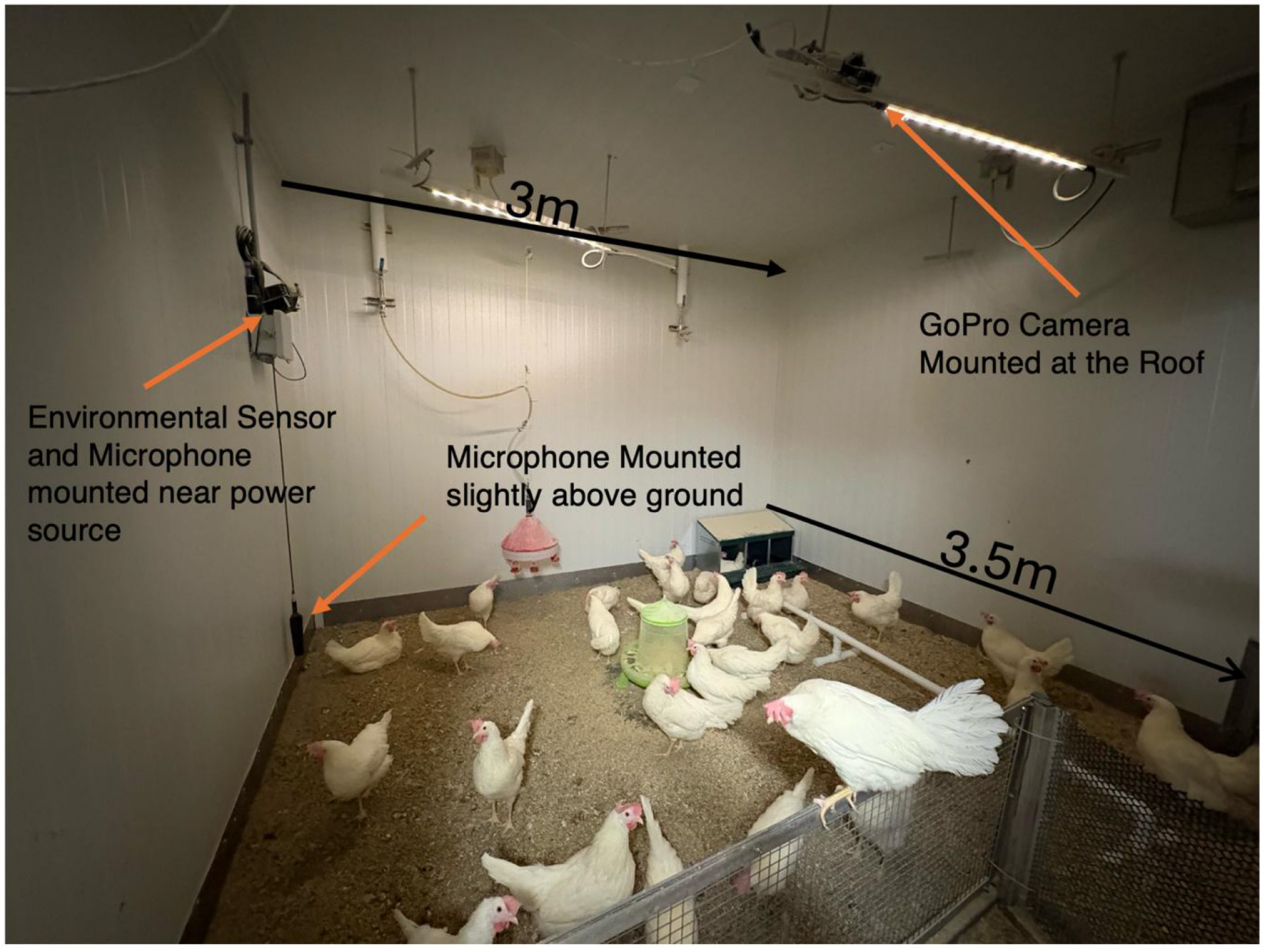
Barn layout and sensor deployment configuration (3 m × 3.5 m). Overhead camera positions, microphone placement (~2 m height), thermal capture zones (~50 cm distance), and environmental sensor location are shown with spatial reference.

Birds were housed in a floor-based system on wood-shaving litter to allow unrestricted locomotion and natural flock behavior. Each barn measured approximately 3 m × 3.5 m. The spatial layout of sensors within the barns is illustrated in Figure 1, highlighting overhead camera placement, microphone positioning, thermal imaging zones, and environmental monitoring points to ensure full spatial coverage and cross-modal synchronization.

Environmental conditions within each barn were regulated using the facility's automated climate control system. Temperature and relative humidity were maintained according to established husbandry guidelines appropriate to the developmental stage of the hens (Ferreira et al., 2024; Kim et al., 2020). Environmental parameters were logged consistently to provide contextual metadata for multimodal data streams and to ensure stable baseline operating conditions during acquisition.

### 2.2 Animal population

A total of 150 Lohmann LSL Lite laying hens were monitored across five barns, with 30 birds housed per barn. The Lohmann LSL Lite strain was selected due to its widespread commercial relevance in egg production systems. Birds were placed in the facility on 5 June 2025 and monitored continuously for 22 consecutive weeks. The extended duration of data acquisition was designed to generate longitudinal multimodal records spanning the full 22 week longitudinal acquisition window. The focus of the present study is the data acquisition framework and infrastructure; therefore, the biological developmental trajectory is referenced only to define the temporal coverage of the dataset.

### 2.3 Ethical approval

All animal procedures were reviewed and approved by the Dalhousie University Animal Care and Use Committee (Dalhousie ACUC), Protocol Approval No. 2025-012, in accordance with the Canadian Council on Animal Care guidelines. The study was conducted as non-invasive observational research. No experimental manipulations were introduced beyond standard husbandry practices. All sensing devices were installed to avoid direct physical contact with the birds, and data collection procedures were designed to minimize disturbance to routine barn operations.

## 3 Multimodal data acquisition framework

The multimodal data acquisition framework was engineered to enable synchronized capture, structured storage, and reproducible processing of heterogeneous sensor streams under commercial-style barn conditions. The system was designed with four guiding principles: non-invasive deployment, temporal synchronization, scalable storage, and downstream computational compatibility.

**Figure 2:**
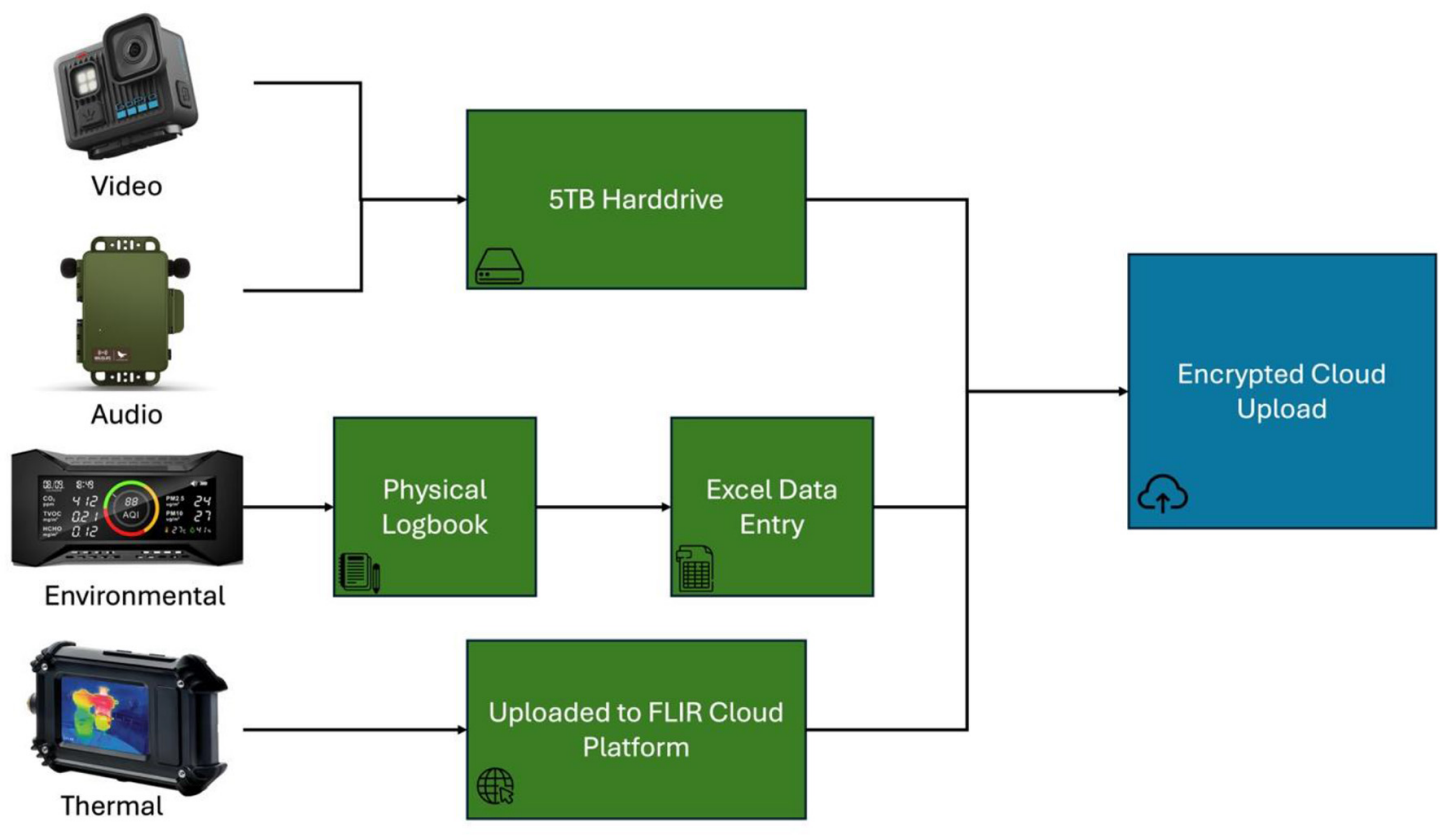
Data acquisition and storage architecture demonstrating synchronized multimodal capture across heterogeneous sensors, enabling reproducible Big Data infrastructure for longitudinal welfare monitoring.

Figure 2 illustrates the acquisition architecture, showing the flow of data from sensor capture to local buffering, scheduled transfer, and centralized storage. The framework integrates four complementary modalities: RGB video, audio, thermal imaging, and environmental sensing. Each modality operates at a distinct sampling frequency and produces data in different formats, requiring explicit coordination at the infrastructure level. Table 1 summarizes the sensor configuration across the five barns. All acquisition components were deployed to minimize disturbance to routine barn operations while maintaining consistent spatial coverage and timestamp integrity.

**Table 1:** Overview of recording sites and multimodal sensor setup.

Barn ID	Animal population	Audio	Video	Thermal	Environmental
1	30 Lohman LSL Lite	Zoom H4N Pro + Rode NTG 2	GoPro Hero 13	FLIR Cx5	Simbow CF-20
2	30 Lohman LSL Lite	Wildlife Acoustic Song Meter SM 4	GoPro Hero 13	FLIR Cx5	Simbow CF-20
3	30 Lohman LSL Lite	Zoom F6 Recorder + Sennheiser MKH 416	GoPro Hero 13	FLIR Cx5	Simbow CF-20
4	30 Lohman LSL Lite	-	GoPro Hero 13	FLIR Cx5	Simbow CF-20
5	30 Lohman LSL Lite	Zoom H4N Pro + Rode NTG 2	GoPro Hero 13	FLIR Cx5	Simbow CF-20

### 3.1 Thermal data acquisition

#### 3.1.1 Hardware specifications

Radiometric thermal images were acquired using a FLIR Cx5 thermal imaging camera capable of capturing per-pixel temperature information in radiometric JPEG format. The rJPEG format preserves embedded temperature metadata, enabling quantitative extraction of region-of-interest statistics during post-processing. Thermal images were stored in both native radiometric format and standard JPEG for compatibility with downstream workflows. Radiometric data were later processed using platform-specific extraction tools to obtain temperature values for defined regions of interest.

#### 3.1.2 Acquisition protocol

Thermal imaging was conducted every two days to balance temporal resolution with manual acquisition constraints. Given this every-two-day sampling schedule, thermal images are intended to provide coarse longitudinal physiological context rather than frame-synchronous input for fusion with 30 fps video. Images were captured at approximately 50 cm distance under consistent angular orientation to reduce geometric variability. Each image was labeled with date and barn identifiers and organized into structured directories to preserve traceability. Because thermal acquisition was manual, strict quality control criteria were applied. Images affected by motion blur, occlusion, or incomplete framing were excluded. Although manual acquisition reduces temporal granularity compared to automated systems, it enables high-fidelity radiometric capture without introducing additional hardware overhead into the barn environment. Thermal imaging is therefore not intended for frame-level fusion with video streams, but provides complementary physiological context that cannot be captured through RGB or audio modalities, particularly for longitudinal assessment of thermal stress and environmental load.

#### 3.1.3 Infrastructure constraints

Thermal data collection presented three primary infrastructure considerations:

- Manual acquisition limited sampling frequency.
- Occlusion and clustering introduced variability in usable images.
- Radiometric file formats required specialized software for metadata extraction.

These constraints informed preprocessing pipeline design and highlight the importance of standardized file organization and documentation in multimodal agricultural datasets.

### 3.2 Video data acquisition

#### 3.2.1 Camera configuration

**Figure 3:**
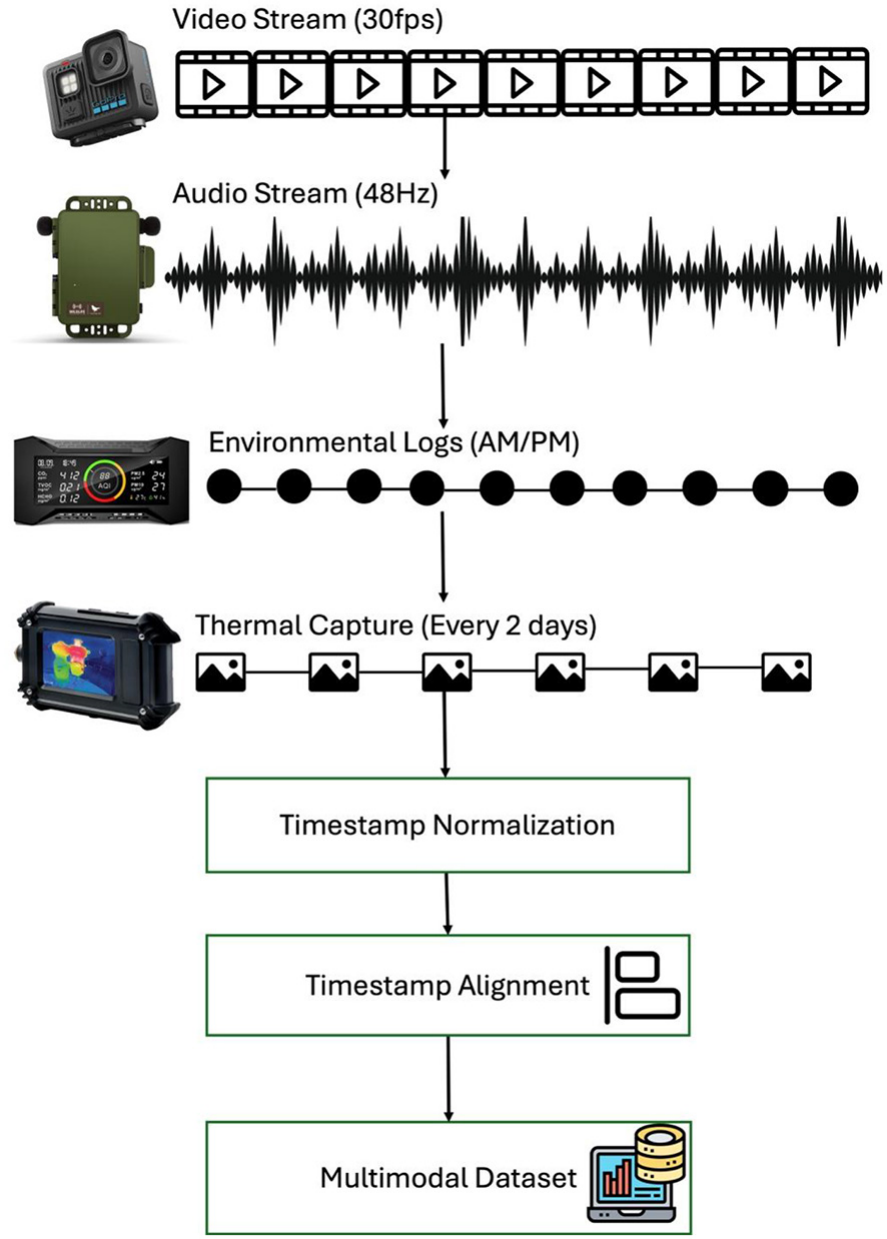
Multimodal temporal synchronization framework. Schematic illustration of heterogeneous sampling frequencies across video (30 fps), audio (48 kHz), periodic thermal imaging, and twice-daily environmental logging, and how timestamp normalization plus fixed alignment windows provide a unified temporal index for downstream multimodal analysis.

Continuous RGB video was captured using overhead-mounted GoPro Hero 13 cameras configured at 1920 × 1080 resolution and 30 frames per second with HEVC compression. Video files were recorded in MP4 format and stored locally on SD cards prior to scheduled transfer. The choice of 1080p resolution reflects a trade-off between spatial detail and storage burden. At 30 fps across five barns, video constituted the largest portion of the dataset, approximately 6 TB of the total 10.2 TB. Artificial barn lighting was consistent across all rooms to maintain uniform lighting conditions. However, to maintain the hens' natural diurnal pattern, the lights were turned off at night. All 30 hens were visible within the cameras' field of view, allowing comprehensive observation of group level and individual level behaviors. Figure 3 illustrates the multimodal synchronization framework, highlighting the heterogeneous sampling frequencies across modalities and the instances where timestamp normalization and fixed alignment windows occur.

#### 3.2.2 Deployment strategy

Cameras were mounted overhead to maximize spatial coverage while minimizing occlusion. Each device was fixed in a stable position to ensure consistent framing throughout the 22 week acquisition period. Lighting conditions followed standard barn schedules. Nighttime recordings were retained despite low visibility to preserve temporal continuity across modalities. This decision supports longitudinal consistency, even if certain segments require illumination correction during preprocessing.

#### 3.2.3 Operational constraints

Video acquisition introduced several infrastructure-level challenges:

- Continuous power management for 24-h recording.
- Intermittent overheating during early deployment stages.
- High storage demand and file transfer scheduling.
- Illumination variability affecting downstream computer vision tasks.

To mitigate overheating, resolution was standardized at 1080 p. Video files were periodically transferred to external hard drives and subsequently uploaded to encrypted cloud storage. No real-time compression beyond HEVC encoding was applied to preserve data fidelity.

### 3.3 Audio data acquisition

#### 3.3.1 Recording systems

**Figure 4:**
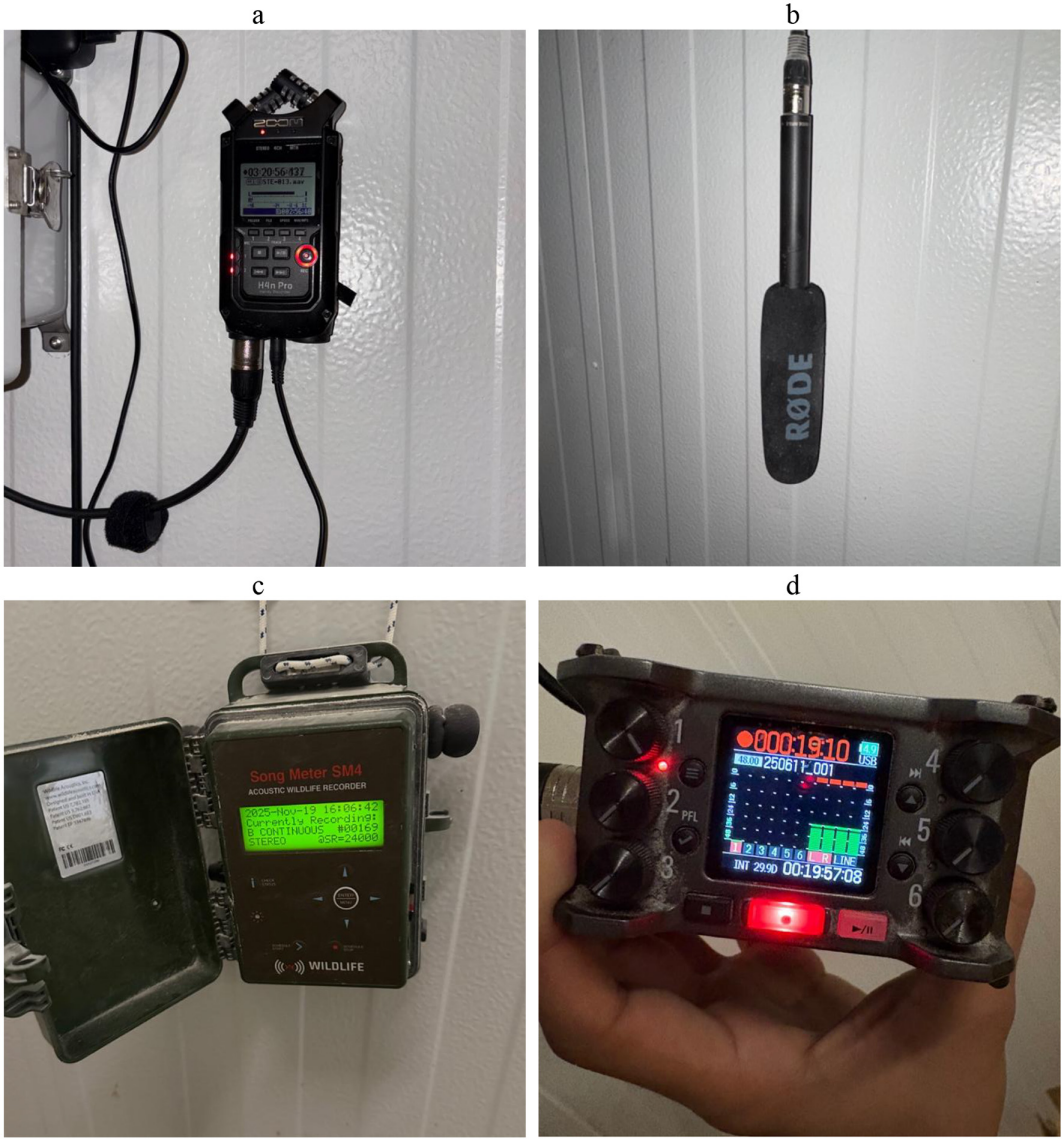
Audio recording instruments deployed in the field: **(a)** Zoom H4 Pro portable recorder, **(b)** Rode NTG 2 microphone, **(c)** Wildlife Acoustic Song Meter SM4 autonomous recorder, **(d)** Zoom F6 multitrack field recorder.

Audio streams were captured using a combination of Zoom H4N Pro recorders, Zoom F6 field recorders, and Wildlife Acoustic Song Meter SM4 units. Devices were configured to record at 48 kHz sampling rate and 24-bit depth in uncompressed WAV format. Table 2 provides technical specifications for each recording system, including frequency response and microphone directivity patterns. The use of multiple as showcased in Figure 4, recorders reflects practical deployment considerations across barns while maintaining consistent sampling parameters.

**Table 2:** Detailed description of audio recording devices.

Room Id	Recording device	Frequency response	Max SPL	Phantom power	Directivity pattern
1	Zoom H4N Pro + Rode NTG 2	Microphone: 20 Hz−20 kHz	Microphone: 131 db SPL	+48 V	Supercardioid
2	Wildlife Acoustic Song Meter SM 4	-	126 db SPL	-	Omnidirectional
3	Zoom F6 Recorder + Sennheiser MKH 416	Microphone 40 Hz−20 kHz	Microphone: 130 db SPL	+48 V	Supercardioid/Lobar (Shotgun)
4	-	-	-	-	-
5	Zoom H4N Pro + Rode NTG 2	Microphone: 20 Hz−20 kHz	Microphone: 131 db SPL	+48 V	Supercardioid

The Zoom F6 Field Recorder (Figure 4d) is a professional field recorder capable of recording 32-bit float recording and possesses a dual AD converter. The Zoom F6 Field recorder was selected due to its capability in capturing both subtle and explosive sounds at full audio quality. Additionally, its rugged nature makes it able to withstand dynamic scenarios like poultry barns. During the data collection process, we attached the Sennheiser MKH 416 microphone to the recorder. The Sennheiser MKH 416 is a short shotgun tube microphone with excellent directivity and compact design. It is highly immune to humidity due to the RF condenser design and can withstand dynamic conditions like the poultry barn. This configuration enables clear capture of poultry vocalizations.

The Wildlife Acoustics Songmeter SM4 (Figure 4c) is an autonomous bioacoustics recorder with weatherproof casing which features two second-generation built-in microphones that create higher quality recordings of birds. Its dual channel feature ensures recording continues even if one microphone is compromised by wildlife, providing redundancy for long term deployments.

The Zoom H4n Pro Handy Recorder (Figure 4a) is a portable four track digital recorder with built in X/Y stereo microphones capable of recording sound pressures up to 140 dB. The H4n supports high quality 24 bit WAV and supports flexible channel configurations, making it suitable for capturing ambient barn sounds and poultry vocalizations. During the data collection process, we attached a Rode NTG 2 Pro Microphone (Figure 4b) to the recorder to enable directional audio with reduced background sound. This configuration allows clear poultry vocalization while minimizing the interference from environmental noise.

#### 3.3.2 Placement and sampling strategy

Microphones were mounted approximately 2 m above floor level to reduce physical interference and floor-level acoustic artifacts. Recording was continuous over the full 22 week period. Each 2.5-h audio segment generated approximately 2 GB of data, resulting in an aggregate audio volume of approximately 4.2 TB. This continuous acquisition model preserves diurnal cycles and rare acoustic events, enabling downstream temporal analysis.

#### 3.3.3 Data engineering considerations

Audio acquisition introduced variability in microphone response profiles across barns. Environmental noise sources including ventilation systems and feeder interactions contributed to background signal complexity. All recordings were timestamped at capture, enabling alignment with video and environmental streams. Post-processing pipelines were designed to address noise reduction while preserving spectral information required for machine learning tasks.

### 3.4 Environmental data acquisition

#### 3.4.1 Sensor specifications

**Figure 5:**
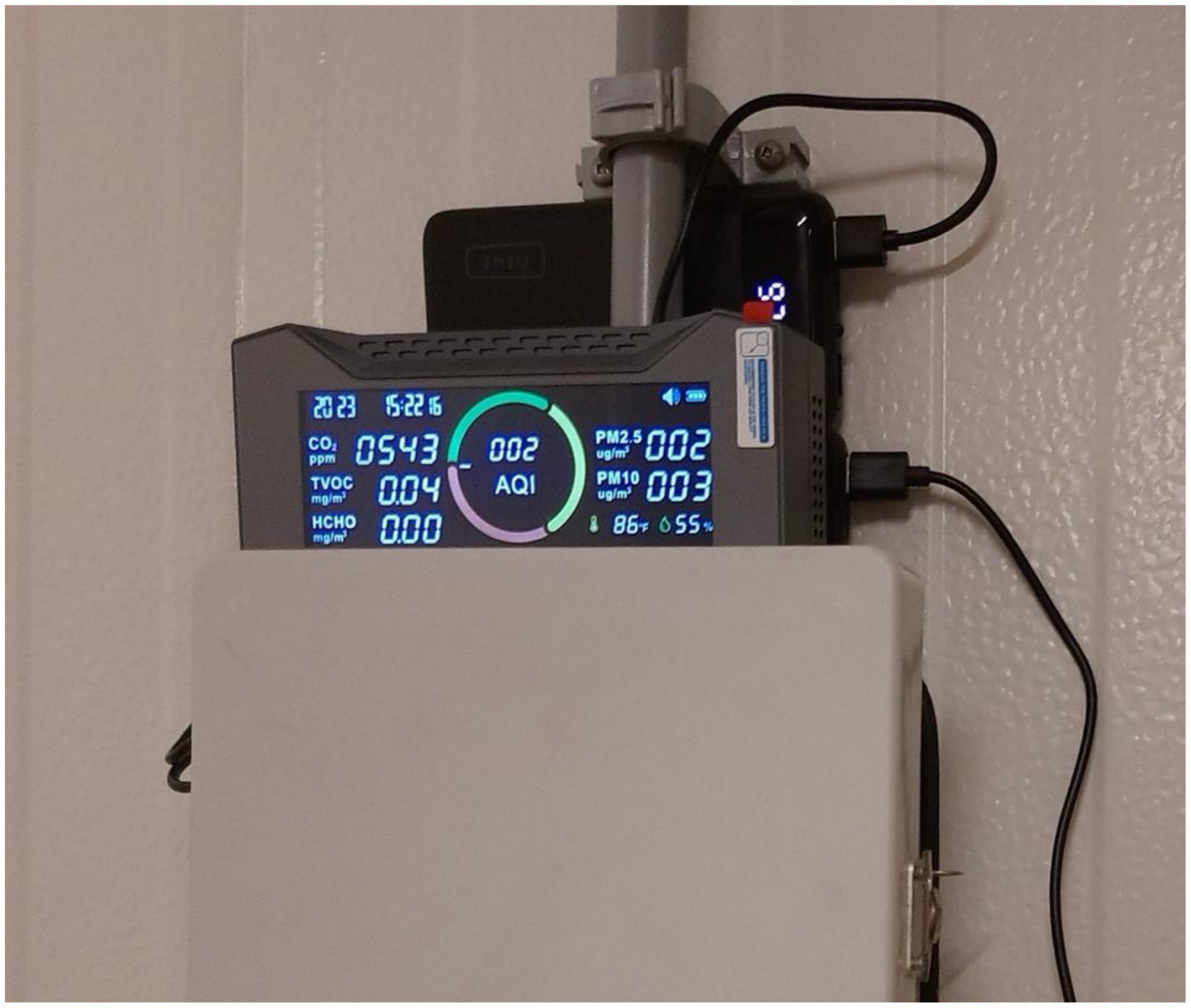
Simbow CF-20 environmental sensor deployment. Wall-mounted configuration positioned to minimize airflow bias and ensure consistent ambient measurements. Environmental logging supports structured contextual metadata capture for temporal synchronization and multimodal dataset alignment across the 22-week acquisition period.

Environmental data were recorded using Simbow CF-20 multi-parameter monitors as shown in Figure 5. Although capable of measuring additional variables such as VOCs and particulate matter, only temperature and relative humidity were consistently logged across the full acquisition period and are included in the present dataset release. Environmental measurements were recorded twice daily and stored in structured spreadsheet formats with timestamp metadata. Twice-daily environmental measurements therefore serve as low-frequency contextual covariates at the barn level, not as continuous signals for instant frame-by-frame fusion.

The CF-20 can capture real time digital measurement and displays it through its large screen which makes it suitable for observers to get accurate readings at a glance. Additionally, the parameters displayed are vital to poultry welfare, respiratory health and behavioral responses. This separation of roles reflects a hierarchical multimodal design, where high-frequency behavioral modalities are complemented by low-frequency physiological and environmental context signals.

#### 3.4.2 Deployment and logging

Sensors were wall-mounted to avoid airflow distortion and remained in fixed positions throughout the study. Environmental readings were manually logged and later digitized into standardized formats. While manual logging limits temporal resolution, it reduces system complexity and supports consistent longitudinal coverage without introducing additional automated hardware dependencies.

### 3.5 Data integrity and storage architecture

Data integrity was maintained through periodic transfer of files from local storage media to centralized repositories. Raw files were preserved in their native formats throughout acquisition.

The storage pipeline followed a three-stage process:

i Local capture and buffering
ii Scheduled transfer to external hard drives
iii Encrypted cloud upload and repository organization

No preprocessing was performed during acquisition. All transformation steps are documented separately to ensure full reproducibility. This structured acquisition and storage strategy enables consistent synchronization across modalities and supports scalable downstream analysis of high-volume heterogeneous agricultural data streams.

### 3.6 Temporal synchronization and alignment

All sensing devices operated with independent internal clocks and were not connected through a shared hardware trigger or centralized timing system. At the start of each recording period, the internal clocks of GoPro cameras and audio recorders were manually synchronized to a common barn reference time. Thermal images and environmental measurements were time-stamped at the point of acquisition by the operator. Cross-modal alignment was therefore performed using recorded timestamps rather than hardware-level clock locking.

The four modalities operate at distinct temporal resolutions. Continuous RGB video was recorded at 30 frames per second, and audio at 48 kHz, while thermal images were acquired every two days and environmental measurements were recorded twice daily. Temporal alignment was performed using fixed temporal windows appropriate to each modality, with video–audio streams aligned at second-level resolution and thermal and environmental streams aligned at hourly to daily resolution. Video and audio streams were aligned using timestamps at frame-level resolution, whereas thermal data were aligned to the nearest recording day and environmental data to predefined AM and PM windows.

Because independent devices were used, residual clock drift between video and audio streams is expected. Based on periodic manual verification, temporal misalignment between video and audio streams is on the order of seconds over daily timescales. Empirically, temporal misalignment between video and audio streams was observed to remain within approximately ±1–2 s over daily recording periods, which is acceptable for behavioral and activity-level analysis but not for fine-grained multimodal fusion.

The dataset is therefore designed for coarse- and medium-scale temporal analysis, longitudinal modeling, and contextual multimodal fusion. It is not intended for applications requiring sub-frame or microsecond-level synchronization across modalities. This design reflects practical deployment constraints in commercial-style agricultural environments, where distributed sensing systems are typically operated independently.

While hardware-level synchronization was not implemented, timestamp-based alignment reflects the operational reality of distributed agricultural IoT systems, where independent sensing devices are deployed without centralized triggering. Similar alignment strategies are widely adopted in field-based monitoring environments and are sufficient for behavioral, contextual, and longitudinal analysis at second-to-minute temporal scales. The dataset is therefore explicitly designed for multi-timescale analysis rather than fine-grained sensor fusion requiring sub-frame precision. This synchronization strategy reflects practical deployment conditions in agricultural IoT systems, where hardware-level clock locking is rarely feasible, and supports reliable behavioral and contextual multimodal analysis at operational timescales.

## 4 Multimodal data preprocessing pipelines

### 4.1 Environmental data preprocessing

Environmental data were consolidated into standardized spreadsheet formats following structured preprocessing steps focused on organization, harmonization, and metadata consistency rather than transformation. Raw Excel files were initially stored separately by room and month. These files were systematically inspected to ensure uniform variable naming conventions, consistent timestamp formatting, and alignment of measurement units across all barns. Subsequently, monthly files were merged into a single longitudinal dataset per room to facilitate temporal continuity and cross-modal synchronization. Missing or undefined values were explicitly encoded as NA or NAN entries to preserve transparency and prevent imputation bias. No smoothing, filtering, normalization, or statistical transformation was applied to the environmental measurements in order to retain raw acquisition characteristics. This conservative preprocessing strategy ensures compatibility with diverse downstream analytical pipelines and supports reproducibility.

### 4.2 Thermal data preprocessing

Thermal images were recorded in radiometric JPEG format, preserving embedded per-pixel temperature metadata. Preprocessing of thermal data primarily involved file organization, quality control, and structured extraction of temperature values from defined regions of interest. Raw rJPEG files were uploaded to the FLIR Ignite platform to enable radiometric temperature extraction. Due to limited open-source support for direct rJPEG parsing, a hybrid workflow was adopted combining vendor-supported extraction with structured tabulation.

**Figure 6:**
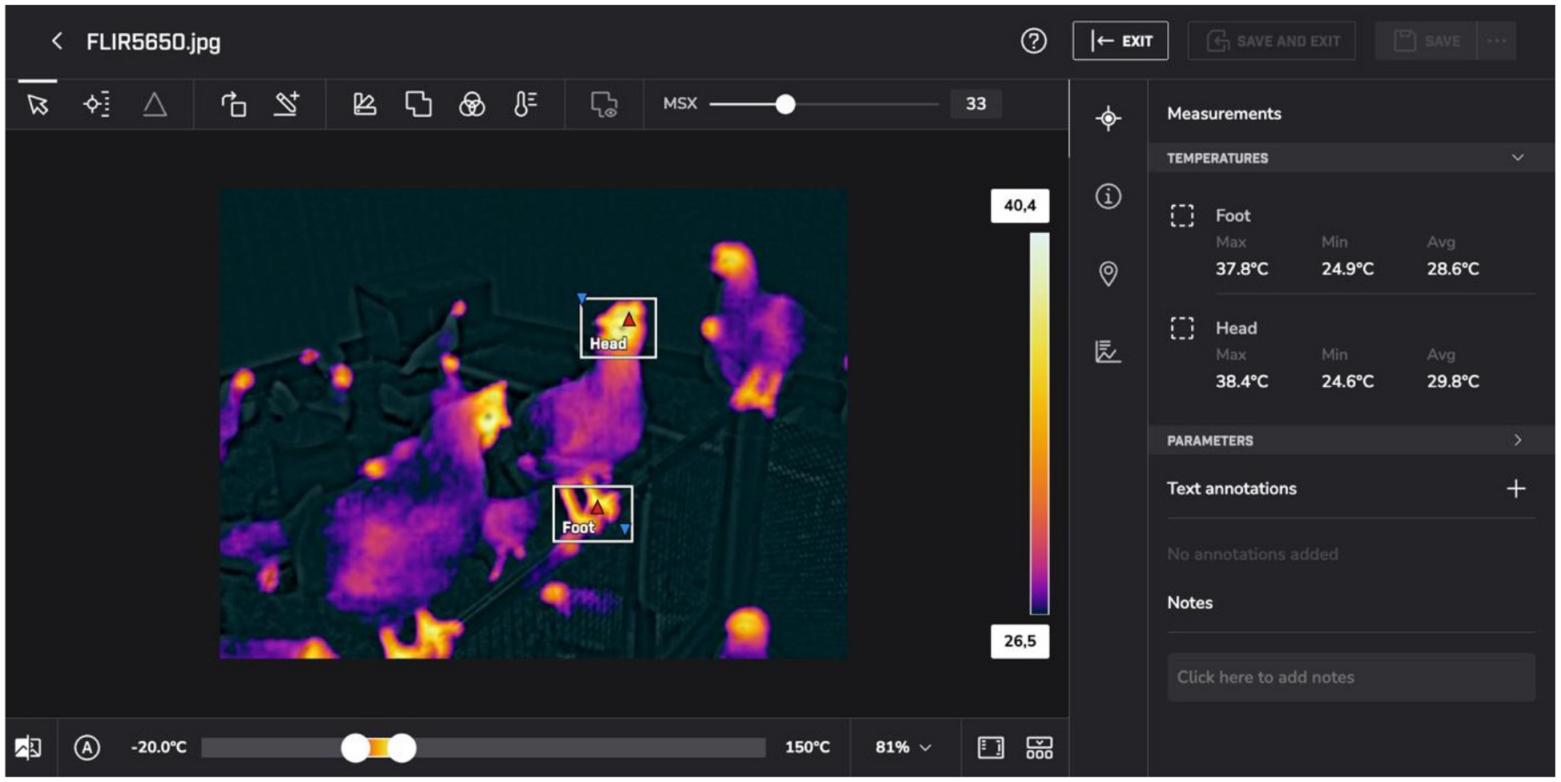
Thermal image segmentation workflow showing manual annotation of thermoregulatory regions (head and foot), enabling quantitative temperature extraction across developmental stages.

Figure 6 illustrates the segmentation workflow used for region annotation and temperature extraction. Images were screened for clarity and absence of occlusion prior to processing. Selected images were grouped by week and barn to maintain chronological ordering. Head and foot regions were manually delineated using platform tools, and minimum, maximum, and mean temperatures were recorded in structured spreadsheets. For early weeks in which specific regions were not reliably visible, values were encoded as NAN or zero where appropriate. This explicit encoding preserves dataset integrity and prevents artificial continuity in longitudinal analyses. No interpolation or temperature normalization was performed at this stage.

### 4.3 Video data processing

Video preprocessing was designed to standardize illumination conditions while preserving original spatial and temporal resolution. Raw MP4 files were retained in their native 1080 p, 30 fps format throughout acquisition. Initial parameter tuning was conducted using visual inspection to identify brightness, contrast, and hue adjustments suitable for low-light barn environments on a single. When identified, all final parameters were then implemented programmatically using a custom Python pipeline built with OpenCV, enabling automated batch processing across large volumes of video files. This uniform, scripted pipeline ensures reproducibility and avoids per-clip tuning.

**Figure 7:**
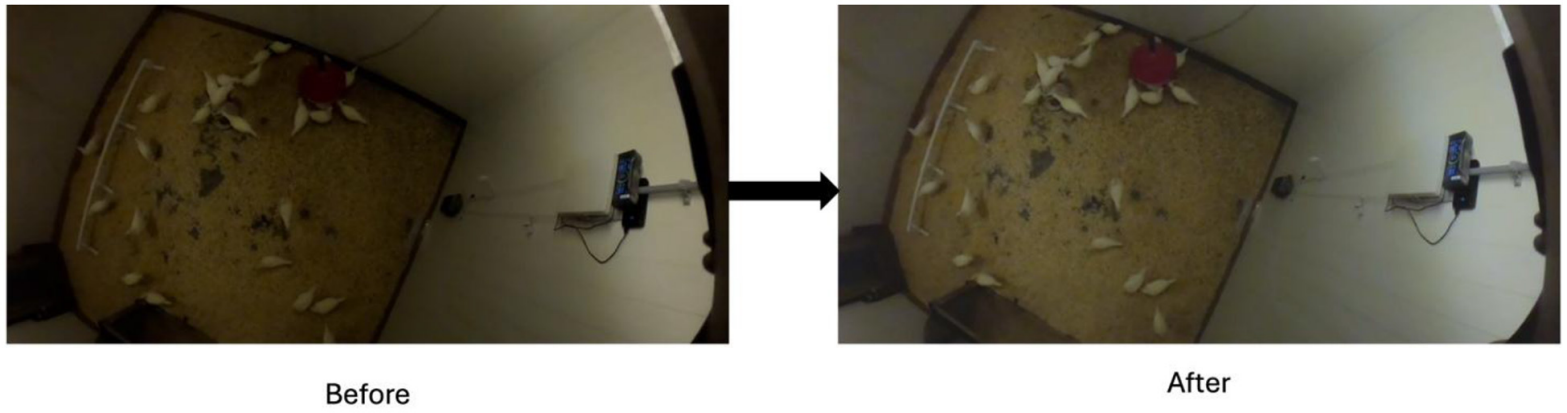
Pre- and post-processing of video data highlighting brightness correction to ensure reliable tracking of hens.

Brightness was increased by 25% through linear scaling, while contrast and hue were adjusted by 10 and 20%, respectively. These values were selected to improve visibility without introducing overexposure or color distortion. Figure 7 presents representative frames before and after preprocessing. Processed videos retained original resolution, frame rate, and compression format. No frame interpolation, cropping, or geometric transformation was applied. Video preprocessing increased the mean frame intensity from 41.96 to 64.48, reflecting a clear improvement in overall visibility. The automated batch approach minimized manual intervention and ensured consistent parameter application across approximately 6 TB of video data. All preprocessing steps were implemented using fully reproducible, scripted pipelines based on open-source libraries including OpenCV and librosa, with no proprietary or manual processing included in the final dataset generation. Quantitative validation showed that preprocessing increased mean frame intensity from 41.96 to 64.48 and improved contrast without altering motion-derived features, confirming that signal enhancement was achieved without distortion of behavior-relevant information.

### 4.4 Audio data processing

Audio preprocessing aimed to reduce background noise while preserving spectral features relevant for downstream analysis. All recordings were stored in uncompressed WAV format at 48 kHz sampling rate and 24-bit depth. Initial noise reduction parameters were calibrated using Audacity through visual inspection of spectrograms and auditory validation. Selected parameters included 12 dB noise reduction, sensitivity level of 12, and frequency smoothing across four bands.

**Figure 8:**
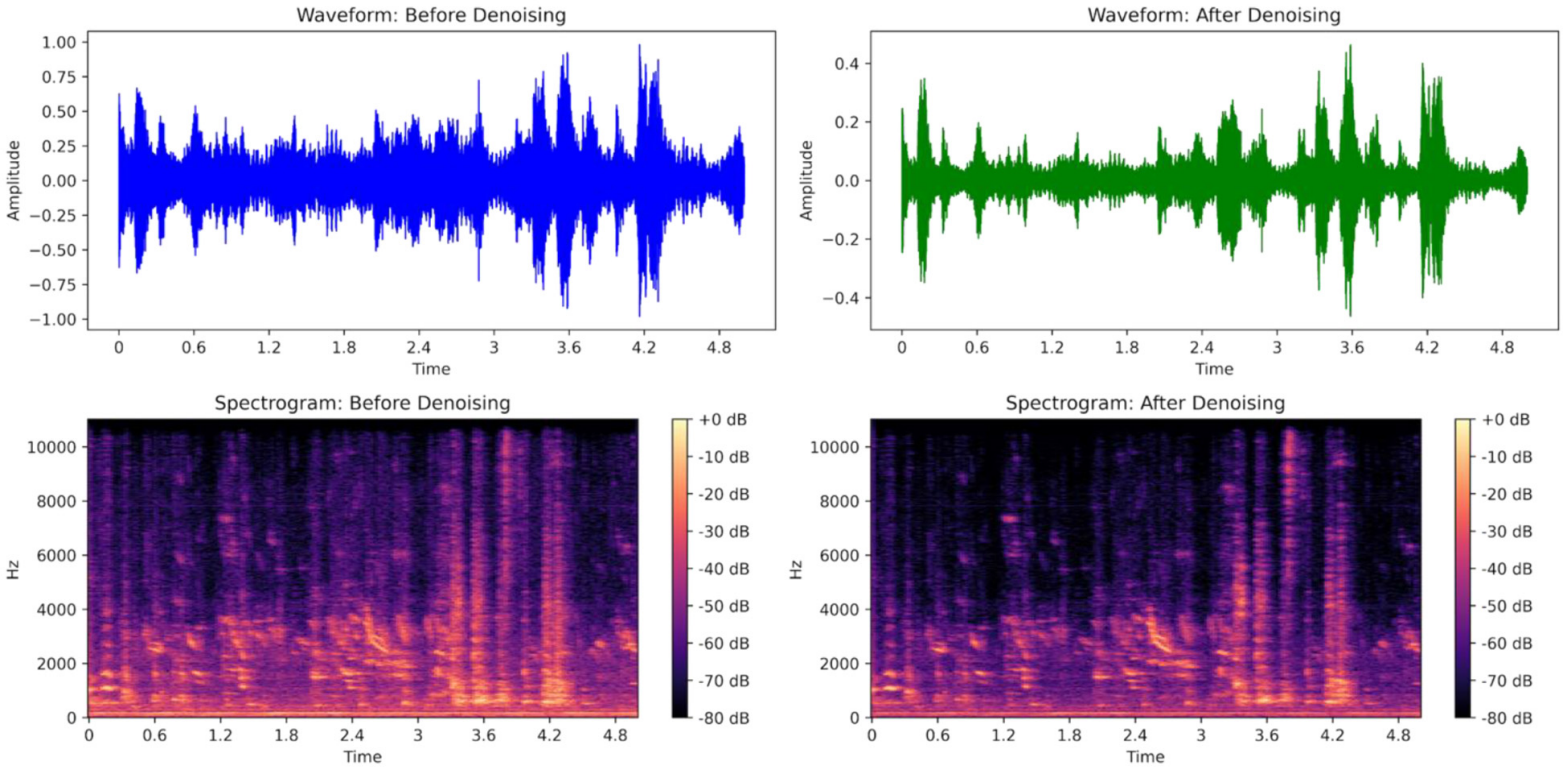
Spectro-temporal representation of audio illustrating effective noise removal while preserving critical vocal features.

These parameters were implemented programmatically using Python-based workflows incorporating the noisereduce library (Sainburg and Zorea, 2025) and librosa (Mcfee et al., 2015). Batch processing enabled automated denoising of large audio volumes while preserving waveform integrity. Figure 8 illustrates representative spectrograms before and after denoising. Noise-reduction parameters were selected to preserve the spectral structure of known vocalization bands while attenuating stationary background noise; the same configuration was applied to all recordings to maintain comparability across barns and weeks. Validation consisted of comparative inspection of waveform amplitude distributions and spectral continuity. No compression, downsampling, or feature extraction was performed during preprocessing to maintain raw signal fidelity for future analytical flexibility.

We assessed the impact of our audio preprocessing pipeline on sample audio clips as demonstrated in Table 3. While absolute SNR values changed due to denoising transformations, spectral characteristics relevant to vocalization analysis were preserved. Spectral analysis showcased that the spectral centroid changed minimally (+39 Hz), and the peak frequency maintain the same value, indicating that our preprocessing pipeline reduces background noise without altering key vocalization characteristics.

**Table 3:** Comparison of audio quality metrics before and after denoising.

Metric	Before denoising	After denoising	Change
SNR (dB)	9.22	5.98	−3.24
Spectral centroid (Hz)	1,631.68	1,670.84	+39.16
Peak frequency (Hz)	171.88	171.88	0.00

The observed reduction in SNR reflects removal of stationary background noise components rather than loss of relevant signal, as evidenced by the preservation of peak frequency and minimal shift in spectral centroid.

## 5 Dataset management and preliminary data exploration

### 5.1 Environmental data exploration

#### 5.1.1 Room level AM and PM summary

To characterize diurnal structure and inter-room consistency, mean morning (AM) and afternoon (PM) temperature and relative humidity values were computed for each barn, as summarized in Table 3. Across all rooms, mean temperatures remained relatively stable between AM and PM intervals, ranging from 24.6 °C to 25.4 °C. In contrast, relative humidity exhibited broader variation, with mean values spanning 43%−55%. The AM and PM measurements demonstrate that environmental logging captured consistent daily patterns while maintaining comparability across barns. Although AM–PM differences were modest, these temporal markers provide structured reference points for multimodal alignment and exploratory analysis.

#### 5.1.2 Descriptive statistics by room

To further characterize distributional properties, descriptive statistics were computed for each barn, including mean, standard deviation, minimum, and maximum values for temperature and relative humidity, as presented in Table 4. Temperature variability across barns was moderate, with standard deviations between 2.1 °C and 3.0 °C. Relative humidity displayed greater dispersion, with standard deviations ranging from approximately 9.75%−15.43%. Minimum and maximum values indicate that environmental conditions spanned a realistic operational range across the 22-week period.

**Table 4:** Room-level AM and PM mean values for temperature ( °C) and relative humidity (%).

Room_ID	Temp_AM	Temp_PM	RH_AM	RH_PM
Room 1	24.70	24.68	48.13	49.35
Room 2	25.17	25.13	43.07	42.88
Room 3	24.62	24.68	52.24	52.79
Room 4	25.28	25.29	52.28	52.72
Room 5	25.31	25.35	54.45	55.48

Table 5 aggregates these statistics across all barns, providing a consolidated overview of environmental stability. The consistency observed across rooms supports the integrity of environmental logging and its suitability for contextual alignment with other modalities.

**Table 5:** Descriptive statistics for environmental variable by room.

	Temp AM				Temp PM			
	Mean	Std	Min	Max	Mean	Std	Min	Max
Room 1	24.70	2.19	21.60	32.0	24.68	2.14	23.5	31.7
Room 2	25.17	2.47	23.29	32.59	25.13	2.47	23.4	32.8
Room 3	24.62	2.57	22.40	31.55	24.68	2.36	23.3	31.6
Room 4	25.27	2.92	22.35	32.80	25.29	2.77	22.5	32.4
Room 5	25.30	2.99	22.35	32.85	25.35	2.82	23.4	32.5
	**RH_AM**				**RH_PM**			
	**Mean**	**Std**	**Min**	**Max**	**Mean**	**Std**	**Min**	**Max**
Room 1	48.13	12.11	26.0	89.0	49.35	13.42	26	84
Room 2	43.07	15.43	15.0	68.0	42.88	15.60	14	77
Room 3	52.24	9.75	40.0	88.0	52.78	11.81	26	82
Room 4	52.29	10.31	35.0	88.0	52.72	13.24	28	83
Room 5	54.45	10.91	21.0	88.0	55.48	12.95	22	85

### 5.2 Thermal data exploration

#### 5.2.1 Descriptive statistics of thermal measurements

Thermal imaging was conducted every two days across the 22 week acquisition period, yielding approximately 3,200 radiometric images suitable for quantitative extraction. Images were captured at standardized distances of approximately 50 cm to maintain geometric consistency. Temperature extraction focused on head and foot regions of interest. Across the full dataset, head surface temperatures ranged from 30 °C to 33 °C, while foot surface temperatures ranged from 25 °C to 30 °C. The consistent difference between central and peripheral measurements, typically 3–5 °C, indicates consistent central and peripheral measurement differentials within the radiometric dataset. These summary statistics provide baseline distributional context for thermal measurements without introducing transformation or normalization, preserving raw acquisition characteristics for downstream modeling. Additionally, these patterns indicate that, despite its sparse sampling, the thermal modality captures smooth longitudinal changes in surface temperature that can be used as physiological context when analyzing behavioral or acoustic changes over weeks. To illustrate cross-modal use, weekly mean head surface temperature was compared with weekly mean ambient temperature from the environmental sensor. The two measures exhibited consistent co-variation across the 22-week period, indicating that the thermal modality captures physiologically meaningful responses to ambient conditions at a coarse temporal scale. This confirms that thermal imaging contributes meaningful physiological signal at coarse temporal resolution, despite its lower sampling frequency relative to video and audio streams.

#### 5.2.3 Temporal structure in radiometric data

**Figure 9:**
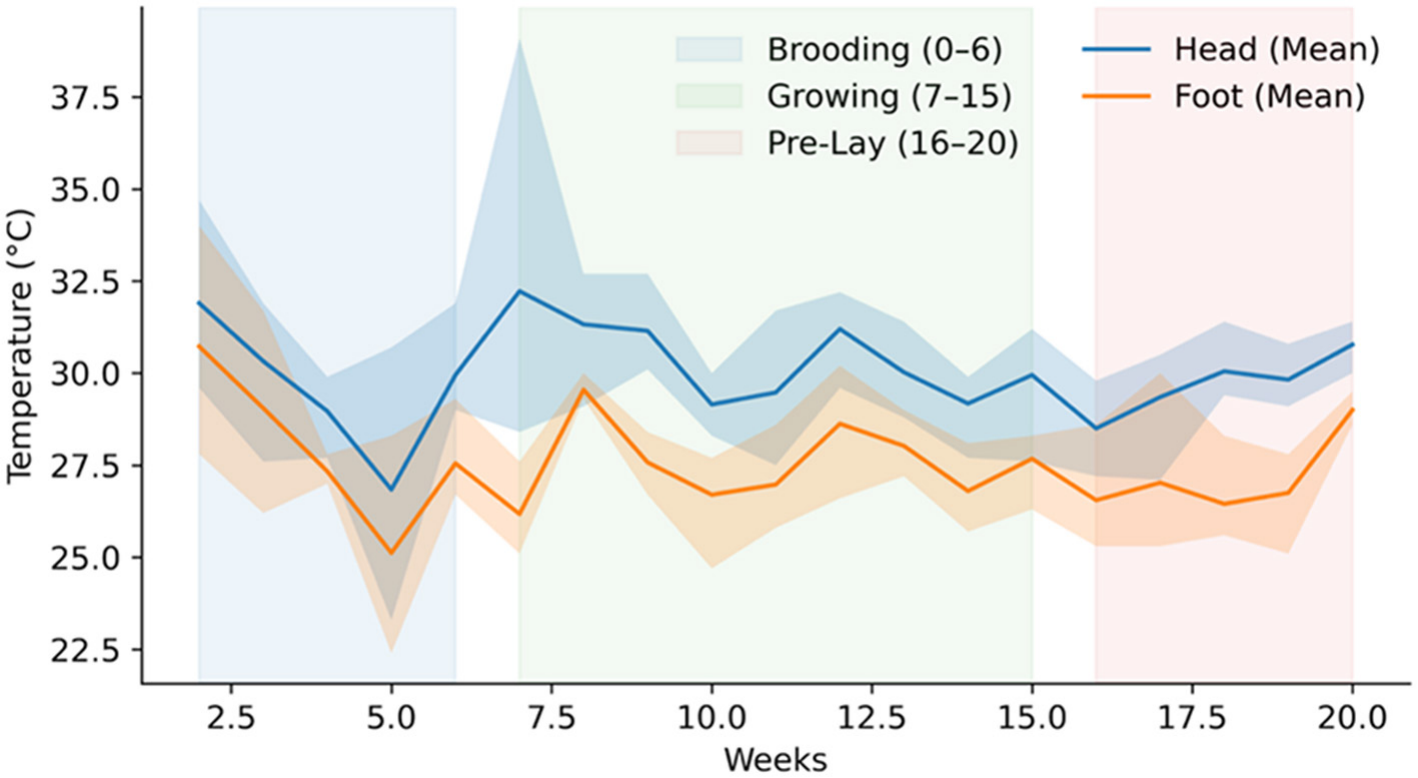
Weekly surface temperatures of head and feet in laying hens, demonstrating maturation of physiological temperature regulation over time.

Weekly aggregation of extracted temperature values, illustrated in Figure 9, reveals structured temporal continuity across the acquisition period. Early weeks exhibit greater variability, followed by progressive stabilization in later intervals. From a data systems perspective, this temporal continuity confirms consistent radiometric capture and annotation across extended duration. The observed reduction in variance over time reflects structured longitudinal signals rather than discontinuities in acquisition or extraction workflows. The parallel trajectories of head and foot measurements further indicate stable region-of-interest extraction and consistent radiometric processing throughout the dataset.

### 5.3 Audio data exploration

Continuous acoustic recording across four barns generated approximately 12,000 h of audio data over the 22 week period. Microphones were mounted at approximately 2 m above floor level to ensure consistent capture geometry and minimize physical interference. Descriptive inspection of audio streams reveals clear diurnal patterns, with lower activity during dark cycles and elevated acoustic density during daytime operational periods. The dataset encompasses a broad range of vocal signatures, including baseline ambient sound, social vocalizations, and event-driven acoustic signals. Following the denoising procedures described in Section 4.4, signal quality remained suitable for spectral analysis and machine learning workflows. The extended temporal coverage supports alignment with synchronized video, thermal, and environmental streams, enabling cross-modal experimentation without additional reformatting.

### 5.4 Video data exploration

Video acquisition was continuous across all five barns, yielding approximately 15,600 h of usable footage due to nighttime low-illumination intervals. Overhead camera placement provided consistent spatial coverage across functional zones within each enclosure. Descriptive inspection confirms the presence of common barn activities including feeding, resting, locomotion, and perching. Early weeks display higher clustering density, while later weeks show more distributed spatial patterns. From an infrastructure perspective, the primary significance lies in the sustained, uninterrupted capture of high-resolution video streams across extended duration. The dataset preserves raw frame structure and temporal continuity, enabling downstream computer vision analysis, behavior modeling, and event detection pipelines without loss of fidelity. Hourly vocalization density derived from the audio streams showed peaks during morning feeding and daytime activity periods, which coincided with peaks in motion-based activity indices computed from the video. Both modalities showed strongly reduced activity during the dark phase, demonstrating consistent circadian structure across streams. This alignment demonstrates the feasibility of cross-modal analysis, where high-frequency behavioral signals from video and audio can be interpreted in conjunction with slower contextual modalities such as thermal and environmental measurements.

### 5.5 Big data characteristics of the dataset

The dataset exhibits defining properties of Big Data across the dimensions of volume, variety, velocity, veracity, and value (Ugochukwu and Phillips, 2024). These characteristics are summarized in Table 6 and discussed below.

**Table 6:** Aggregated descriptive statistics of environmental variable across all rooms.

	Mean	Std	Min	Max
Temp_AM	24.97	2.59	21.6	32.85
Temp_PM	24.97	2.48	22.5	32.80
RH_AM	49.57	12.61	15.0	89.0
RH_PM	50.20	14.11	14.0	85.0

#### 5.5.1 Volume

The 22 week acquisition process generated approximately 10.2 TB of raw multimodal data. Video constituted the largest share, approximately 6 TB, corresponding to 15,600 h of footage at 1080 p and 30 fps. Audio streams contributed approximately 4.2 TB, representing 12,000 h of continuous recordings at 48 kHz. Thermal imaging comprised roughly 3,200 radiometric JPEG files, and environmental logging contributed approximately 3,000 structured entries. This scale necessitates structured storage strategies, periodic transfer scheduling, and batch preprocessing pipelines. The dataset size supports large-scale training experiments while preserving temporal continuity.

#### 5.5.2 Variety

The dataset integrates four heterogeneous modalities with distinct sampling frequencies and file formats. Video is stored in compressed MP4/HEVC format at 30 fps. Audio is stored in uncompressed WAV format at 48 kHz. Thermal images are preserved in radiometric JPEG format. Environmental data are stored in structured CSV and XLSX formats. This heterogeneity requires explicit synchronization and modality-specific preprocessing workflows. The diversity of data types enables multimodal fusion research while simultaneously increasing alignment complexity.

#### 5.5.3 Velocity

Video and audio streams were recorded continuously, generating uninterrupted temporal data streams across five barns. Environmental measurements were recorded twice daily, and thermal images were acquired every two days. The sustained duration of 22 consecutive weeks captures extended temporal variation, diurnal cycles, and rare events. Continuous capture ensures that temporal modeling approaches can operate without artificial segmentation or synthetic interpolation.

#### 5.5.4 Veracity

Real-world barn environments introduce structured noise, occlusion, illumination variability, and manual logging artifacts. Video streams contain low-light segments during dark cycles. Audio recordings include ventilation noise and overlapping vocalizations. Thermal images occasionally exhibit occlusion due to flock density. Environmental logging involves manual transcription steps. Rather than filtering these constraints at acquisition, the dataset preserves them as authentic operational features. Such preservation enhances ecological validity and supports development of noise-robust AI systems. Across the 22 week window, effective video coverage represented approximately 98% of planned recording hours, primarily due to early overheating and power interruptions, as summarized in Table 7. Audio completeness exceeded 98%. Of 3,200 captured thermal images, ~87% passed quality filters. Environmental logs achieved 98% completeness across barns. All missing observations are explicitly encoded using NA/NAN markers in the released files.

**Table 7:** Big data characteristics and technical specifications.

Dimension	Characteristic	Specification	Implication for AI research
Volume	Total dataset size	10.2 tb	Requires large storage and batch processing strategies
	Video data	~6 tb, ~15,600 h @ 1080 p, 30 fps	Enables large scale behavior model training
	Audio data	~4.2tb, ~12,000 h @ 48 kHz	Enables acoustic classification and vocal repertoire analysis
	Thermal images	500 mb - ~ 3200 paired FLIR-native and radiometric JPEG files	Enables radiometric temporal modeling.
	Environmental data	100 mb	Provides contextual metadata for multimodal alignment.
Variety	Modality types	4 (video, audio, thermal, and environmental)	Sufficient for multimodal fusion architecture
	Sampling rates	30 fps (video), 48 kHz (audio), periodic (thermal and env)	Requires temporal resampling for synchronization
	File formats	MP4/HEVC, WAV, rJPEG, CSV/XLSX	Requires format specific preprocessing.
Velocity	Acquisition mode	Continuous 24/7 (audio and video) AM and PM (environmental) Every 2 days (thermal)	Captures diurnal patterns and rare events
	Collection duration	22 consecutive weeks	Showcases longitudinal production phases.
	Full 22-week acquisition window.	Pullet to adult	Cross-modal temporal learning
Veracity	Real world constraints	Low-light, occlusion, noise, clustering, manual data logging	Allows real world deployment but requires data preprocessing
	Missing data	-	Requires missing data handling strategies
	Video completeness	~15,288 h usable of ~15,600 h planned (~98%)	Gaps due to early overheating; all missing intervals encoded as NA
	Audio completeness	~12,350 h usable of ~12,600 h planned (>98%)	High continuity across 22-week period
	Thermal completeness	2,800 of ~3,200 images passed quality filters (87.5%)	Occlusion and motion blur excluded *via* quality control
	Environmental completeness	98% of expected entries recorded across barns	Missing values explicitly encoded as NA/NAN
Value	Supported tasks	Behavior recognition, multimodal fusion, anomaly detection, temporal modeling	Multipurpose research applications
	Novel capabilities	Longitudinal multimodal modeling, cross modal learning	Allows explorations to new research directions
	Deployment relevance	Commercial style barns, non-invasive	Production-scale deployment relevance

#### 5.5.5 Value

The dataset functions as multipurpose research infrastructure supporting computer vision, acoustic classification, multimodal fusion, temporal modeling, and anomaly detection workflows. The integration of synchronized environmental metadata enhances contextual modeling. Beyond model training, the dataset provides a documented blueprint for large-scale multimodal acquisition in agricultural systems. By combining open access, reproducible preprocessing pipelines, and explicit infrastructure documentation, this work advances scalable data engineering practices for precision livestock applications.

The five Big Data dimensions interact to amplify system complexity. Volume increases the likelihood of noise accumulation. Variety requires explicit temporal alignment strategies. Velocity preserves rare and transient events while increasing storage and transfer burden. Together, these properties distinguish the dataset as a production-scale multimodal resource and highlight the operational realities that agricultural AI systems must address.

## 6 Discussions

### 6.1 Research significance and contribution

The rapid expansion of precision livestock farming has intensified interest in sensor-driven monitoring frameworks capable of operating at commercial scale (Fan et al., 2025; He et al., 2025; Neethirajan, 2020). Yet much of the current literature remains modality-specific, emphasizing algorithmic performance within constrained data environments. Video analytics, acoustic classifiers, thermal imaging, and environmental sensing are frequently developed in isolation. Such fragmentation limits the capacity of deployed systems to capture complex, temporally evolving conditions within real production settings.

This study contributes at the level of infrastructure rather than model optimization. By designing and documenting a synchronized acquisition framework integrating RGB video, audio, thermal imaging, and environmental sensing across 22 consecutive weeks, we address foundational constraints in multimodal data engineering. Managing 10.2 TB of heterogeneous data requires explicit coordination of storage architecture, timestamp integrity, modality-specific preprocessing, and long-duration data transfer scheduling. The technical challenge lies not only in capturing data, but in preserving coherence across asynchronous streams and ensuring reproducibility at scale.

The framework demonstrates that sustained multimodal acquisition in commercial-style barns is feasible when supported by structured preprocessing pipelines and systematic quality control. Each modality captures distinct signal characteristics, and the value of integration lies in complementarity rather than redundancy. However, the primary contribution is infrastructural. The dataset functions as a reusable backbone upon which diverse analytical strategies can be constructed. It enables multimodal temporal representation learning, cross-modal alignment research, missing-modality robustness experiments, and unsupervised anomaly detection under realistic noise conditions. From a multimodal AI perspective, the four streams play complementary roles. Overhead RGB video resolves spatial distribution and group-level movement patterns. Audio captures vocal signatures associated with social interaction and stress. Thermal imaging provides lower-frequency physiological context in the form of surface temperature gradients. Environmental sensing contributes structured contextual metadata about barn-level conditions. The infrastructure is therefore designed for fusion architectures that treat video and audio as fast channels and thermal and environmental measurements as slower contextual channels.

Importantly, this work shifts emphasis from narrow predictive benchmarks toward sustainable data ecosystems. By releasing raw multimodal streams alongside documented preprocessing workflows, the study promotes methodological transparency and lowers barriers for subsequent machine learning research. The dataset is not tied to a single hypothesis or classifier. Instead, it provides a platform for exploratory and hypothesis-driven modeling across a broad range of AI architectures.

The broader implication is that multimodal data infrastructure, rather than individual sensing devices, represents the limiting factor in next-generation agricultural AI systems. Longitudinal coherence, storage resilience, and synchronization logic determine whether multimodal learning can transition from laboratory prototypes to production-scale deployment.

### 6.2 Practical challenges and ecological validity

Data acquisition in agricultural environments cannot be decoupled from operational realities. Lighting conditions optimized for animal management constrain video visibility. Flock density introduces occlusion and variable spatial resolution. Ventilation systems generate persistent acoustic background noise. Manual logging of environmental variables introduces human-dependent latency. These conditions are not aberrations but intrinsic features of commercial systems.

From a systems perspective, preserving these conditions within the dataset strengthens ecological validity. Artificially sanitizing noise, occlusion, or variability during acquisition would produce cleaner signals at the expense of realism. Instead, the dataset retains these complexities, requiring downstream models to confront authentic deployment constraints. Robust AI systems must operate within such imperfect environments.

Audio acquisition illustrates this tension clearly. Overlapping vocalizations and environmental noise complicate signal isolation. While denoising pipelines enhance clarity, complete disentanglement of individual acoustic sources remains unresolved. This highlights a key insight: multimodal fusion is not merely additive but disambiguating. Video streams can provide spatial context to acoustic events, while environmental data can contextualize signal variability. Integration therefore mitigates limitations inherent in any single modality. This complementarity allows downstream models to integrate high-velocity behavioral information with slower contextual variables, which aligns with typical multimodal fusion paradigms in Big Data and AI systems.

Similarly, illumination variability required post-processing adjustments in video streams. Rather than treat these adjustments as corrective measures alone, they reflect the iterative relationship between sensor deployment and computational refinement. Infrastructure and analytics co-evolve. In this sense, preprocessing is not ancillary but integral to multimodal system design.

### 6.3 Alignment with sustainable development goals and digital infrastructure

The study contributes directly to SDG 9 by advancing digital infrastructure within agricultural production systems. Sustainable innovation in livestock sectors depends not only on sensing hardware but on interoperable data architectures, transparent workflows, and scalable storage strategies. By prioritizing deployable, non-invasive sensors and documenting end-to-end acquisition protocols, this work emphasizes reproducibility over proprietary optimization.

Open release through Zenodo and GitHub transforms the dataset from a local experiment into a shared research resource. Publicly accessible multimodal datasets reduce duplication of effort and facilitate comparative benchmarking across modeling strategies. They also enable cross-institutional collaboration and replication, both essential for maturing precision agriculture into a robust scientific domain.

The inclusion of synchronized environmental metadata further positions the dataset within climate-aware agricultural analytics. As climate variability intensifies, modeling approaches must integrate environmental context alongside behavioral and physiological signals. Infrastructure that supports such integration is a prerequisite for resilient food production systems.

### 6.4 Limitations and future directions

The limitations of this study arise from the practical constraints of deploying multimodal sensing systems in commercial-style agricultural environments and define the appropriate scope of downstream use.

Temporal synchronization was achieved using timestamp alignment across independently operating devices, without hardware-level clock locking. While manual synchronization to a common reference time was performed, residual drift cannot be fully eliminated. Alignment between continuous modalities such as video and audio is therefore reliable at the scale of seconds, whereas alignment with thermal and environmental measurements is inherently coarser, at the level of hours or days. Accordingly, the dataset is intended for longitudinal analysis and contextual multimodal fusion rather than frame-exact or microsecond-level sensor fusion.

A second limitation arises from heterogeneous sampling frequencies across modalities. Continuous video and audio capture high-resolution behavioral dynamics, while thermal imaging (every two days) and environmental measurements (twice daily) provide lower-frequency physiological and contextual signals. These slower modalities are most appropriately treated as temporally aggregated covariates, particularly within hierarchical or sequence-based modeling frameworks.

The stocking density of 30 hens within a 3 m × 3.5 m enclosure reflects institutional animal-care requirements and controlled research conditions for stable sensing deployment. While higher-density commercial systems may exhibit increased occlusion and more complex interaction dynamics, the multimodal sensing architecture, data acquisition framework, and synchronization strategy are independent of stocking density and remain transferable to commercial environments. Accordingly, the dataset should be interpreted as a methodological benchmark for system design and multimodal integration, rather than as a direct behavioral proxy for high-density production settings.

The dataset is released without manual annotations of behavior, health, or welfare states. This design prioritizes raw signal fidelity and flexibility for diverse analytical tasks but limits immediate use for supervised learning. Instead, the dataset is well suited for self-supervised learning, representation learning, and exploratory multimodal analysis.

Future work will focus on extending the infrastructure through automated environmental logging, hardware-synchronized timing mechanisms, and enhanced low-light or infrared imaging. Additional efforts will target scalable annotation strategies, including expert-labeled subsets, event-driven weak supervision, and semi-automated labeling based on learned representations. These developments will enable the transition from a general-purpose multimodal data infrastructure to task-specific benchmarking resources.

More broadly, this work highlights a shift in agricultural AI from model-centric development toward data-centric system design. Multimodal sensing becomes effective only when supported by coherent, longitudinal, and well-documented data infrastructures. By demonstrating such an infrastructure under real-world conditions, this study provides a foundation for the development of robust, context-aware AI systems in precision livestock farming.

The primary contribution of this work is therefore not fine-grained multimodal fusion at sub-frame resolution, but the design and validation of a scalable, real-world multimodal data infrastructure under operational agricultural constraints. This distinction is critical, as it positions the dataset as enabling robust multimodal AI development within realistic deployment conditions rather than idealized laboratory settings.

## 7 Conclusions

Multimodal sensing in livestock systems is often discussed conceptually, yet practical deployment remains constrained by data engineering realities. Sustained acquisition across heterogeneous modalities demands more than sensor installation. It requires structured synchronization, scalable storage, disciplined preprocessing, and transparent documentation capable of supporting reproducible downstream analysis.

The framework presented here demonstrates that continuous multimodal capture across RGB video, audio, thermal imaging, and environmental sensing can be maintained over extended durations under commercial-style barn conditions. The resulting 10.2 TB dataset reflects not only signal diversity, but also the operational constraints inherent to real agricultural environments. Illumination variability, acoustic interference, occlusion, manual logging, and asynchronous sampling rates are preserved as authentic system characteristics rather than artificially suppressed artifacts. This preservation is intentional. Robust artificial intelligence systems must ultimately perform under such imperfect conditions.

Beyond raw data volume, the primary contribution lies in the articulation of a scalable acquisition and preprocessing architecture. This dataset and associated pipelines are intended as shared infrastructure for the community to develop and benchmark multimodal AI methods under realistic farm conditions. By documenting file formats, synchronization strategies, batch processing pipelines, and storage workflows, the work advances methodological transparency within precision agriculture. Infrastructure, rather than isolated model performance, increasingly defines the ceiling of progress in multimodal AI. Without longitudinal, heterogeneous, openly accessible datasets, sensor fusion remains a theoretical ambition rather than an operational reality.

The open release of synchronized multimodal streams alongside reproducible preprocessing code establishes a foundation for diverse analytical paradigms. Researchers may pursue multimodal representation learning, cross-modal alignment, anomaly detection, or robustness testing without being constrained by predefined outcome labels. In doing so, the dataset functions as shared infrastructure rather than a single-purpose benchmark. Limitations remain and are instructive. Manual environmental logging constrains temporal granularity. Low-light conditions affect certain video segments. Thermal acquisition relies on periodic capture rather than continuous automation. These constraints delineate clear avenues for future system refinement, including hardware-synchronized timestamps, automated environmental logging, and enhanced low-light imaging strategies.

The broader implication extends beyond poultry systems. As agriculture transitions toward data-intensive management, the challenge shifts from sensing feasibility to infrastructural integrity. Scalable multimodal frameworks must balance realism with reproducibility, heterogeneity with coherence, and openness with operational practicality. The architecture described here contributes to that transition by demonstrating that longitudinal, high-volume, multimodal datasets can be generated, curated, and shared without sacrificing ecological validity. Sustainable progress in precision livestock farming will depend on interoperable data ecosystems capable of supporting adaptable artificial intelligence systems. Establishing such ecosystems requires deliberate attention to acquisition design, preprocessing rigor, and open dissemination. The framework and dataset presented here represent a step toward that objective, providing durable infrastructure upon which future multimodal analytics and deployment-ready AI systems can be built.

## Data Availability

The curated dataset and associated metadata are deposited in Zenodo under restricted access (https://doi.org/10.5281/zenodo.18735885), with access provided through a Data Access Agreement to protect farm confidentiality. Metadata, documentation, and code are publicly available, and the open- source preprocessing and feature extraction pipeline is available at https://github.com/mooanalytica/poultry-multimodal-data-infrastructure. The current release contains raw and preprocessed multimodal signals and metadata, but no manual behavior or welfare labels; users are encouraged to develop task-specific annotation layers on top of the provided infrastructure.
